# Efficacy, safety and side effects of noninvasive mechanical ventilation (NIV) in an internal medicine ward

**DOI:** 10.1186/2197-425X-3-S1-A173

**Published:** 2015-10-01

**Authors:** A Puleo, C Argano, D Lucia, D Volpes, I Lo Burgio, MISI NIV Team

**Affiliations:** UOC Medicina Interna Semi-Intensiva. AO Villa Sofia-Cervello Palermo, Palermo, Italy; UOC Medicina Interna. AO Villa Sofia-Cervello Palermo, Palermo, Italy

## Introduction

Over the last two decades, NIV has become the cornerstone in the management of acute respiratory failure (ARF) in ICU. Benefits from NIV have been reported from RCTs and meta-analyses in selected patients admitted to centers with extensive experience. The generalizability of these benefits in non referral centers and in elderly patients with co-morbidities has to be demonstrated.

## Objectives

We retrospectively analyzed the data of patients admitted to our sub-intensive internal medicine department to assess NIV indications, safety, tolerability and success rate.

## Methods

From 1/2013 to 12/2014 critically ill patients presenting with ARF and requiring ventilatory support were included. ARF etiologies were classified into three groups: group A (acute on chronic respiratory failure), group B (cardiogenic pulmonary oedema), and group C (de novo respiratory failure i.e. hypoxemic ARF patients either immunocompetent or immunocompromised).

## Results

Clinical records of 104 patients (6O males, 44 females, mean age 76 years, range 60-94) receiving NIV were reviewed. Group A included 62 patients, group B encompasses 22 subjects and group C comprised 20 persons (mostly pneumonia). All patients had at least 2 co-morbidities either cardiovascular or metabolic disease. Nurses reported poor NIV tolerance in 25 % of patients. Patients self-reported on anxiety in 24 % of the cases, and 9 % complained of an important nose and mouth dryness. Conjunctivitis, nose skin ulcerations or gastric distension were found in 8 % of patients. Nurses also ranked sleep quality as poor in 32 % of patients. NIV success was 69 % overall (95 % CI 65-73 %). Among 34 patients who failed NIV, 20 were submitted to endotracheal intubation and referred to ICU, 14 died.

## Conclusions

In most elderly and chronic-ill patients physicians have difficulties to take decisions when oxygen supplementation and the usual medical therapy are not effective. In these subjects the indication for mechanical ventilation may be questionable. Our data suggest that NIV could be an alternative option as it could provide an effective and safe treatment in a general ward. The difficulty lies in making a proper selection of these patients, how to establish who needs to be admitted in an ICU setting and who could be treated with NIV in a setting of sub-intensity of care. To address this question we need to develop more accurate prediction rules focused on patients' overall prognosis to better select and help families in the decision-making process, and to make a more efficient use of the resources. The major limitations of our study are its retrospective nature, its low power due to the limited sample size, and the lack of a randomized control group. Our data suggest that the use of NIV in internal medicine sub-intensive units is a safe and effective option in the treatment of ARF in elderly patients with co-morbidities, reducing the need of intubation and unnecessary admissions to ICUs.

